# Pharmacological targeting of CXCL12/CXCR4 signaling in prostate cancer bone metastasis

**DOI:** 10.1186/s12943-016-0552-0

**Published:** 2016-11-03

**Authors:** M. Katie Conley-LaComb, Louie Semaan, Rajareddy Singareddy, Yanfeng Li, Elisabeth I. Heath, Seongho Kim, Michael L. Cher, Sreenivasa R. Chinni

**Affiliations:** 1 Department of Urology, Wayne State University, School of Medicine, 9245 Scott Hall, 540 E. Canfield Avenue, Detroit, MI 48201 USA; 2 Department of Pathology, Wayne State University, School of Medicine, 9245 Scott Hall, 540 E. Canfield Avenue, Detroit, MI 48201 USA; 3 Department of Oncology, Wayne State University, School of Medicine, 9245 Scott Hall, 540 E. Canfield Avenue, Detroit, MI 48201 USA; 4 Biostatics Core, Karmanos Cancer Institute, Wayne State University, School of Medicine, 9245 Scott Hall, 540 E. Canfield Avenue, Detroit, MI 48201 USA

**Keywords:** CXL12, CXCR4, Src, EGFR, HER2, Plerixafor, Gefitinib, Bone metastasis

## Abstract

**Background:**

The CXCL12/CXCR4 axis transactivates HER2 and promotes intraosseous tumor growth. To further explore the transactivation of HER2 by CXCL12, we investigated the role of small GTP protein G_αi2_ in Src and HER2 phosphorylation in lipid raft membrane microdomains and the significance of CXCR4 in prostate cancer bone tumor growth.

**Methods:**

We used a variety of methods such as lipid raft isolation, invasion assays, an in vivo model of intratibial tumor growth, bone histomorphometry, and immunohistochemistry to determine the role of CXCR4 signaling in lipid raft membrane microdomains and effects of targeting of CXCR4 for bone tumor growth.

**Results:**

We determined that (a) CXCL12/CXCR4 transactivation of EGFR and HER2 is confined to lipid raft membrane microdomains, (b) CXCL12 activation of HER2 and Src is mediated by small GTP proteins in lipid rafts, (c) inhibition of the CXCL12/CXCR4 axis through plerixafor abrogates the initial establishment of tumor growth without affecting the growth of established bone tumors, and (d) inhibition of EGFR signaling through gefitinib leads to inhibition of established bone tumor growth.

**Conclusions:**

These data suggest that lipid raft membrane microdomains are key sites for CXCL12/CXCR4 transactivation of HER2 via small GTP binding protein G_αi2_ and Src kinase. The initial establishment of prostate cancer is supported by the endosteal niche, and blocking the CXCL12/CXCR4 axis of this niche along with its downstream signaling severely compromises initial establishment of tumors in the bone microenvironment, whereas expanding bone tumors are sensitive only to the members of growth factor receptor inhibition.

**Electronic supplementary material:**

The online version of this article (doi:10.1186/s12943-016-0552-0) contains supplementary material, which is available to authorized users.

## Background

Prostate cancer (PC) is the most commonly diagnosed solid malignancy in men, with over 192,000 new cases yearly, and the second leading cause of cancer-related death in men in the US, with over 27,000 deaths each year. PC has a specific propensity to metastasize to bone; in fact, in the majority of cases, bone metastases develop long before metastatic growth is apparent in soft viscera. Bone metastases cause pain, compression fractures, spinal cord compromise and other complications. Bone metastasized cancer cells induce bone turnover by recruiting bone resident osteoclasts and osteoblasts, and the resultant bone turnover enhances tumor growth by creating a vicious cycle [1]. Cancer cells use similar mechanisms as hematopoietic stem cells in homing to bone by competing for the occupation at osteoblastic niches in bone tissue [2], where chemokine signaling plays a key role in attracting cells to the bone microenvironment [2–4].

The CXCL12/CXCR4 axis has been involved in homing of breast [5] and prostate [4, 6] cancer cells to bone where cancer cells have aberrant expression of CXCR4, the receptor for the CXCL12 chemokine [7–9]. Transcriptional regulation of the CXCR4 gene is a key determinant of net cell surface expression of CXCR4 and its subsequent function in transformed epithelial cells and cancer cells. We showed that TMPRSS2-ERG fusions regulate CXCR4 expression in prostate tumors; thus, androgen induced ERG expression transcriptionally regulates CXCR4 expression in PC cells [7, 9]. In addition, several factors and organ microenvironments have been shown to regulate CXCR4 expression in tumor cells [10–17]. The mammary fat pad and bone microenvironment have been shown to induce CXCR4 gene expression in cancer cells [16, 18]. At the cellular level, osteoblasts, stromal cells and endothelial cells all express CXCL12 [4, 6, 10, 19] and contribute to bone metastasis of PC cells [4, 6].

CXCR4 expression increases during progression of PC; localized prostate carcinoma and bone metastasis tissue express significantly higher levels than benign prostate tissue [20, 21]. Among PC patients, higher expression of CXCR4 was documented in prostate tumor tissues from African Americans [22], suggesting aggressive phenotypes often associated with higher CXCR4 expression. CXCR4 expression is associated with shorter progression free survival in cancers [23], and in prostate cancers its expression is significantly associated with metastatic disease and poor survival [24, 25]. The chemokine CXCL12 is also over-expressed in PC metastatic tissue compared to normal tissue [20]. The CXCL12/CXCR4 axis has been shown to play an important role in PC cell proliferation, migration and invasion [4, 6, 20, 26–30].

Previously, we showed that activation of the CXCL12/CXCR4 axis transactivates HER2 [3, 6] and promotes intraosseous tumor growth [3]. To further explore the transactivation of HER2 by CXCL12, we investigated the role of the small GTP protein G_αi2_ in Src and HER2 phosphorylation in lipid raft membrane microdomains and the significance of CXCR4 inhibition by plerixafor, a bone stem cell mobilizer, in prostate cancer bone tumor growth. Given the important role of CXCL12/CXCR4 signaling in PC bone metastases, our data suggest that CXCL12/CXCR4 inhibition may impact the development of bone metastasis.

## Methods

### Cell culture

Cell lines were cultured in a humidified incubator with 5 % CO_2_ at 37 °C. All media were supplemented with 2 mM glutamine, 100 units/ml penicillin, and 100 μg/ml streptomycin (Life Technologies Inc., Carlsbad, CA). PC3 cells maintained in RPMI-1640 supplemented with 10 % fetal bovine serum (FBS), PC-3 M-luc2 cells in EMEM medium supplemented with 10 % FBS, and C4-2B cells, in T-Medium supplemented with 10 % FBS. All cell lines were authenticated with STR analysis (Genomics core at Michigan State University, East Lansing, MI) and shown to have markers respective for each cell line as established by ATCC and also tested for mycoplasma contamination prior to use with Venor-GeM mycoplasma detection kit (Sigma Biochemicals, St. Louis, MO).

### Western blot analysis

Cells were washed with PBS, and total cellular proteins were extracted with buffer containing 62.5 mM Tris–HCl (pH 6.8), 2 % SDS, 1 mM PMSF, and 1X Protease inhibitor cocktail (Roche, Indianapolis, IN). Protein content was quantified with a BCA protein assay (Pierce Biotechnology, Rockford, IL), and equal amounts of protein were resolved by 10 % SDSPAGE. Immunoblot was performed with antibodies against pHER2 (Y1248) (Catalog # A00318-100, GenScript, Piscataway, NJ), total HER2 (Catalog #, SC-284, Santa Cruz Biotechnology, Dallas, TX) pEGFR (Y1173) (Catalog # 4407 s, Cell Signaling, Beverly, MA), total EGFR (Catalog # 4267 s, Cell Signaling, Beverly, MA), Flotillin (Catalog # 610383, BD Biosciences, San Jose, CA), β-tubulin (Catalog # SC-5274, Santa Cruz Biotechnology, Dallas, TX), pSrc (Catalog # 2101 s, Cell Signaling Technologies, Beverly, MA), total Src (Catalog # 2109 s, Cell Signaling Technologies, Beverly, MA), G_αi2_ (Catalog # SC-7276, Santa Cruz Biotechnology, Dallas, TX), pAkt (S473) (Catalog # 9271 s, Cell Signaling, Beverly, MA), Akt (Catalog # 2938 s, Cell Signaling, Beverly, MA), and GAPDH (Catalog #SC-25778, Santa Cruz Biotechnology, Santa Cruz, CA). The band intensities were determined by quantitation of pixel intensities using ImageJ software (version 10.2; National Institutes of Health, Bethesda, MD).

### Cell fractionation

A successive detergent solubilization method for isolating lipid rafts was previously described [3] and detergent free preparation of cell lysate and density gradient centrifugation was followed as per Macdonald et al. [31].

### Invasion assay

For cells to be treated with Dasatinib (Catalog # D3307, LC Laboratories, Woburn, MA), C4-2B cells were plated on the upper chamber of Matrigel-coated transwell filters (2 × 10^5^ cells/filter) in growth media, supplemented with 1 % FBS, containing 0.5 μM Dasatinib or vehicle control. For Pertussis toxin (PTX) (Catalog # 516561, Calbiochem, La Jolla, CA) studies, cells were pretreated with 200 ng/ml PTX for 3 h prior to cell invasion studies. For cells to be treated with Src siRNA, C4-2B cells were transfected with scrambled or Src siRNA using Lipofectamine 2000 (Invitrogen) 24 h prior to seeding 2 × 10^5^ cells in serum free medium on Matrigel coated inserts. For both Dasatinib and siRNA conditions, cells were allowed to invade for 24 h in the presence or absence of 200 ng/mL CXCL12 added to the bottom chamber. Cotton swabs were used to remove non-migrated cells from the upper chamber, and inserts were stained with 0.9 % crystal violet. Total number of migrated cells was counted under 10X magnification in five fields. Assays were performed in triplicate. *: *p* < 0.05; **: *p* ≤ 0.005. For protein analysis, cells were treated with Dasatinib or Src siRNA as performed for the invasion assays, in the presence or absence of CXCL12. Cell lysate was collected after 24 h and analyzed by Western blot.

### In vivo studies and tumor tissue analyses

PC-3 M-luc2 cells were injected intratibially on Day 0 and saline control or plerixafor treatment was started via an osmotic pump (Alzet, Cupertino, CA). Plerixafor is obtained from Genzyme Corporation and administered in the animal model using an osmotic pump at the rate of 0.5 μl per hour at 20 mg/ml concentration. After 21 or 23 days, tumor growth was determined by in vivo luciferase imaging. For treating of established tumors, mice were sacrificed and ex vivo x-ray imaging of tumor-bearing tibiae was performed at 22 or 24 days post-injection. For treating established tumors, tumors were imaged at day 17, and, based on luciferase signals, tumors were randomly divided into two groups: plerixafor vs saline (control); plerixafor or saline pumps were then implanted at 18 days post tumor cell implantation in tibiae. Further luciferase imaging was performed at day 21 to monitor tumor growth. Mice were sacrificed and ex vivo x-ray imaging of tumor-bearing tibiae was performed at 26 days post-injection. C4-2B cells were infected with lentiviruses expressing luciferase to generate C4-2B-luc cells and stable cells were selected with puromycin treatment. For gefitinib study animals imaged at 15 days post tumor cell injection were randomized as control and treatment groups. Gefitinib (Catalog # G-4408, LC Laboratories, Woburn, MA) is formulated in 0.5 % Tween 80 and administered through oral gavage at 200 mg/kg body weight. Animals were imaged at 23, 29 and 40 days and x-rays, obtained at the 40th day. Luciferase imaging was performed with either Kodak in vivo imager or Carestream in vivo Xtreme imager (Bruker, Bellerica, MA).

### Immunohistochemistry

Formalin-fixed, paraffin-embedded serial tissue sections from control or plerixafor treated bone tumors were deparaffinized with xylene and rehydrated in graded ethanol. Endogenous peroxidase activity was blocked by incubating in 3 % H_2_O_2_ for 20 min; antigen retrieval was performed with proteinase K (Sigma-Aldrich, St. Louis, MO). Slides were then blocked with Blocking Serum from ABC Vectastain Kit (Vector Labs, Burlingame, CA). Slides were incubated at 4 °C overnight in a humidified chamber with antibodies directed against CXCR4 (R&D Systems, Minneapolis, MN) or Ki67 (BD Biosciences, San Jose, CA). After washing, sections were incubated with ABC Vectastain Kit, according to manufacturer’s protocol, followed by incubation with 3, 3-diaminobenzidine tetrahydrochloride (Vector Laboratories, Inc., Burlingame, CA). Nuclei were counterstained with Mayer’s hematoxylin (Sigma-Aldrich, St. Louis, MO). Sections were then dehydrated with graded EtOH, washed with xylene, and mounted with Permount (Sigma-Aldrich, St. Louis, MO). Hematoxylin and eosin staining was also performed on bone tumor sections, and histomorphometric analyses were performed as previously described [3] to determine tumor burden, cortical bone area, and trabecular bone area.

### Statistical analyses

Data were analyzed using GraphPad Prism software and Microsoft Excel 2008. All data are presented as mean ± SE. The in vivo luciferase imaging was performed with two different machines (Kodak invivo imaging (old) and Bruker in vivo Xtream (new) equipment) in case of Figs. 4b, c and 5b , c. Therefore, the expression levels of photons generated by the old machine were normalized by those by the new machine using their geometric means as follows. Suppose there are n and m photon expression levels generated by old and new machines, respectively (i.e., X_1,_ *x*_2_, …, *x*_*n*_; *y*_1,_ *y*_2_, …, *y*_*m*_). Then the *i* th expression level *x*_*i*_ for the old machine will be normalized by ${\overline{x}}_i={x}_i\cdot {\left({\prod}_{k=1}^m{y}_k\right)}^{\frac{1}{m}}/{\left({\prod}_{j=1}^n{x}_j\right)}^{\frac{1}{n}},$ where *i* = 1, 2, …, *m*.

Statistical comparisons were performed using Wilcoxon rank sum test and a *p*-value < 0.05 was considered statistically significant.

## Results

### CXCL12/CXCR4 axis transactivates EGFR members in lipid raft membrane microdomains

**Fig. 1:**
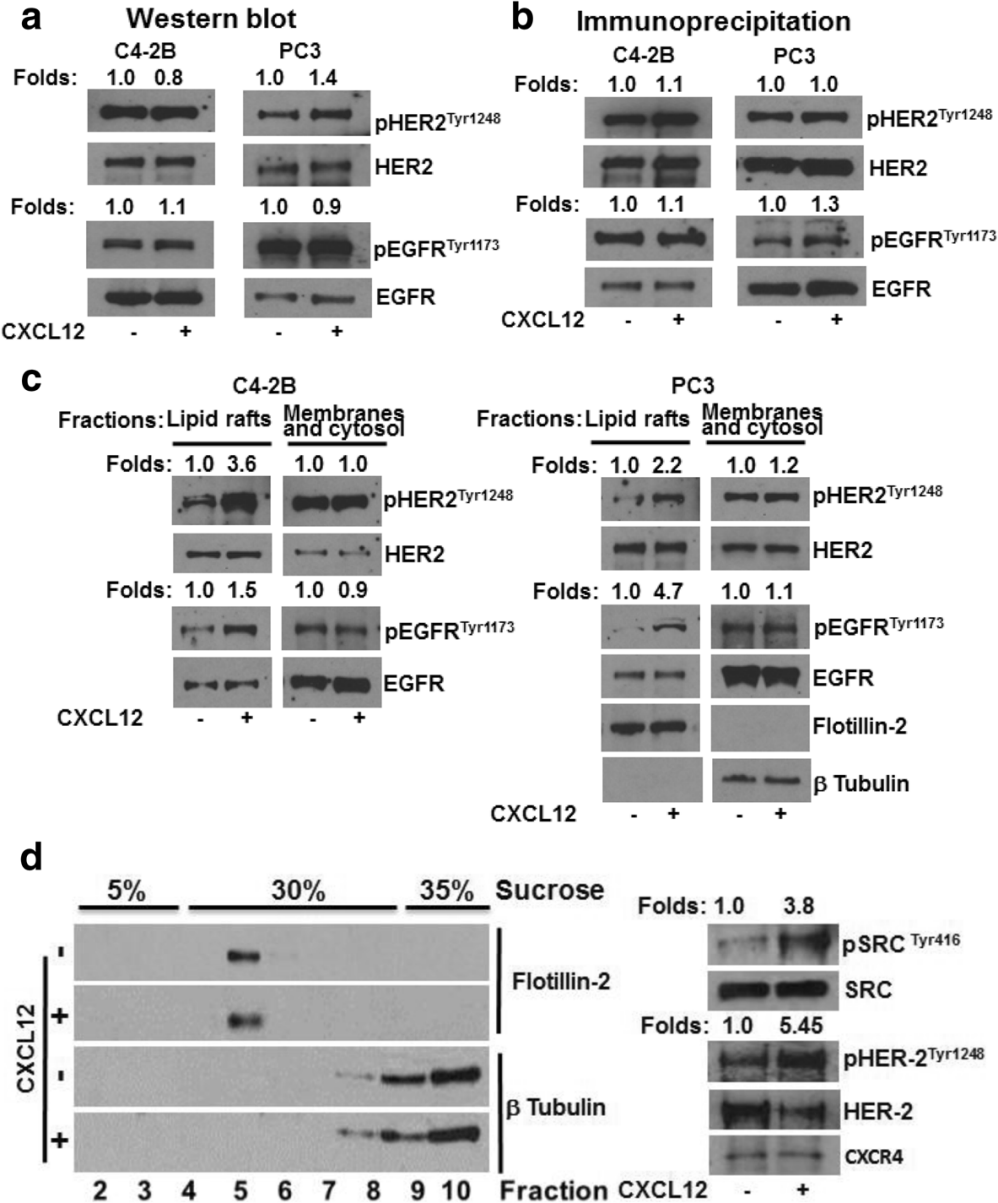
CXCL12/CXCR4 transactivation of growth factor receptors occurs in lipid raft microdomains. **a** C4-2B and PC3 cells were cultured in the presence or absence of CXCL12. Total cell lysates were analyzed for phosphorylated and total HER2 and EGFR. **b** C4-2B and PC3 cells were cultured in the presence or absence of CXCL12. Cell lysates were immunoprecipated with anti-HER2 and EGFR antibodies and immunoblotted with total and phosphorylated HER2 and EGFR antibodies. **c** C4-2B and PC3 cells were cultured in the presence or absence of CXCL12. Lipid raft membrane microdomains were isolated using a successive detergent solubilization method; both lipid rafts and cellular membranes and cytosol fractions were immunoblotted with phosphorylated and native HER2 and EGFR antibodies. **d** PC3 cells were lysed in a detergent-free lysis buffer and fractionated by sucrose density gradient centrifugation. Fractions were immunoblotted with lipid raft marker Flotillin-2 and cytosol marker β-Tubulin. The lipid raft enriched fraction (#5) was immunoblotted with HER2 and phosphorylated HER2 antibodies

Previously, we have shown that CXCL12 signaling is capable of transactivating HER2 in the lipid rafts domains in PC3 cells [3]. In an effort to determine if this transactivation is confined to lipid raft membrane microdomains or occurs elsewhere in the cell and if this transactivation was limited to HER2, we performed experiments with C4-2B and PC3 cells and investigated CXCL12/CXCR4 induced phosphorylation of HER2 and EGFR. Western blot analysis demonstrated that treatment with CXCL12 did not significantly alter phosphorylation of HER2 or EGFR (Fig. 1a). Furthermore, immunoprecipitation of HER2 and EGFR also did not show changes in phosphorylation of either HER2 or EGFR upon treatment with CXCL12 in either C4-2B or PC3 cells (Fig. 1b). On the contrary, a successive detergent solubilization method of lipid raft isolation showed that CXCL12 had indeed induced phosphorylation of both HER2 and EGFR in PC3 and C4-2B cells (Fig. 1c). Our previous studies used detergents to solubilize cells for lipid raft preparation by sucrose density gradient centrifugation, and to avoid detergent induced artifacts in cellular signaling in lipid raft preparation [32], we avoided detergents for cell lysis and prepared lipid rafts using sucrose density gradients. However, upon fractionation, increased levels of pHER2 and pSrc were still found, in the lipid raft fraction of both control and CXCL12 treated PC3 cells (Fig. 1d). These data demonstrate that CXCL12/CXCR4 transactivates both HER2 and EGFR, that this transactivation occurs exclusively in lipid raft microdomains, confirming our previous results, and that detergents in raft preparation do not significantly affect the growth factor receptor transactivation.

### G_αi_ proteins mediate CXCL12/CXCR4 induced Src and HER2 phosphorylation

**Fig. 2:**
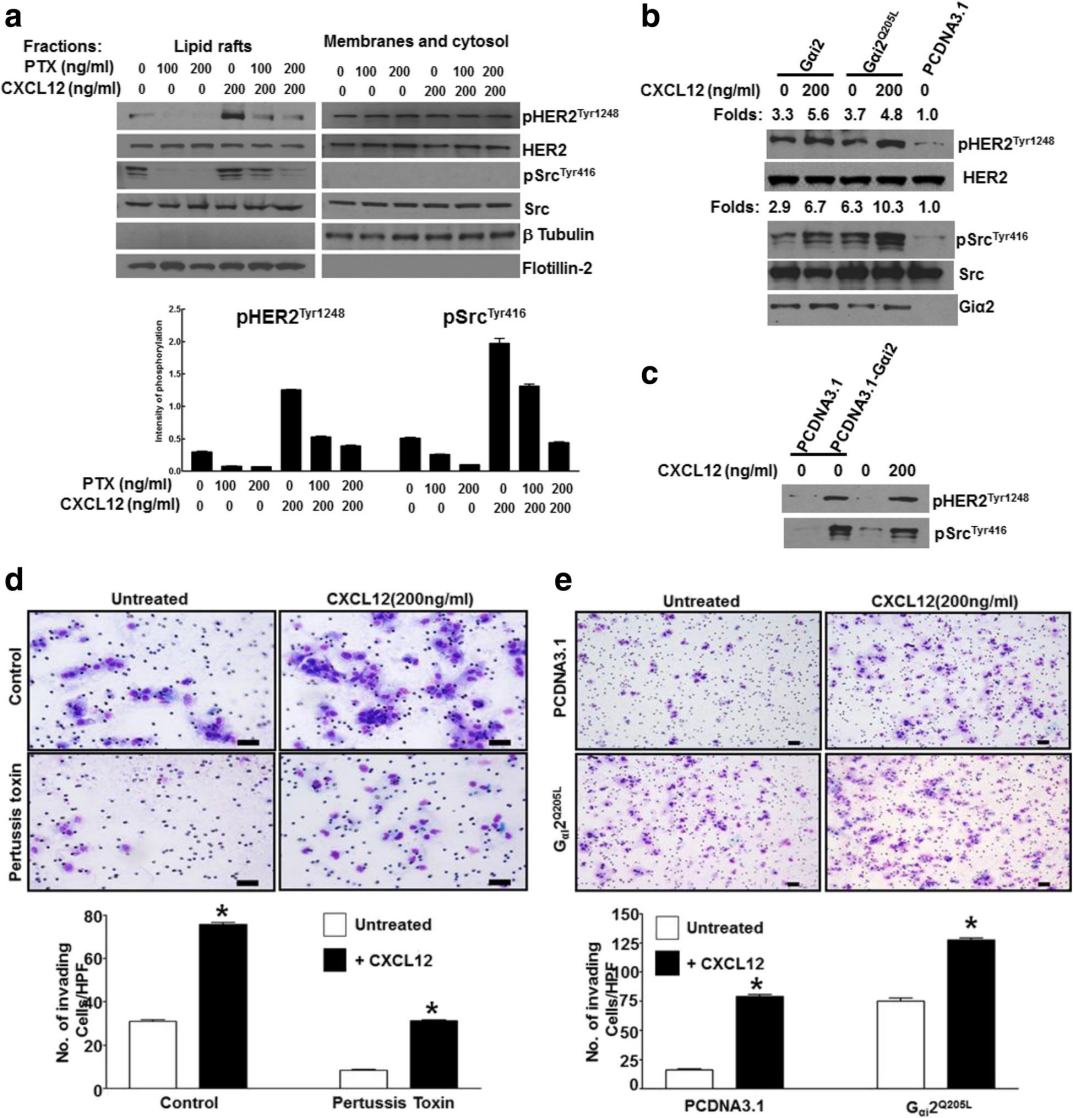
CXCL12 activation of HER2 and Src is mediated by G_αi_ GTP proteins in lipid raft membrane microdomains and this activation induces cell invasion. **a** C4-2B cells were cultured in the presence or absence of increasing concentrations of pertussis toxin (PTX) for 3 h, followed by treatment with CXCL12 for 15 min. Cell lysate was collected, and protein expression of phosphorylated and total HER2 and Src was determined by Western blot analysis. Flotillin was utilized as a loading control for the lipid raft fraction; β-Tubulin was utilized as a control for the membranes and cytosol fractions. Bottom panel shows the quantitation of phosphorylated HER2 and Src. **b** C4-2B cells were transfected with wild type G_αi_ (WT-Gαi2), constitutively active G_αi_ (Q205L), or plasmid control (pcDNA3.1). Cells were then cultured in the presence or absence of CXCL12 for 15 min, and cell lysate was collected. Protein expression of phosphorylated and total HER2 and Src as well as G_αi_2 were determined by Western blot analysis. **c** C4-2B cells were transfected with plasmid control or constitutively active G_αi_ (Q205L) and cultured in the presence or absence of CXCL12. Lipid raft fractions were collected and immunoblotted with phospho HER2 and Src antibodies. **d** C4-2B cells were either treated or not, with PTX (200 ng/ml) and a chemoinvasion assay was performed in the presence or absence of 200 ng/ml CXCL12. **e** C4-2B cells were transfected with pcDNA3.1 or pcDNA3.1 G_αi_2^Q205L^ plasmids and a chemoinvasion assay was performed in the presence or absence of 200 ng/ml CXCL12. Bottom panels show the quantitation of invaded cells. Experiment was performed in triplicates; a representative field of invading cells are shown (**d** and **e**). Number of invading cells was quantitated in three independent experiments and statistical analyses were performed using ANOVA; significance was calculated using Tukey Posttest analysis using GraphPad Prism software. *P* value <0.05 is shown as a *

Members of small G-proteins were shown to promote prostate cancer cell invasion [33–36]. To determine the requirement of heterotrimeric G_αi_ proteins in CXCL12/CXCR4 induced HER2 phoshosporylation and cellular invasion, we treated C4-2B cells with pertussis toxin (PTX) for 3 h to inhibit G_αi_ proteins (Fig. 2) and this treatment did not affect the cell viability. Pertussis toxin treatment leads to inhibition of basal phosphorylation of both HER2 and Src in lipid raft membrane microdomains (Fig. 2a). To determine the effect of PTX on CXCL12 dependent HER2 and Src phosphorylation, cells were pretreated with PTX followed by CXCL12 activation. PTX treatment inhibited CXCL12 activated HER2 and Src phosphorylation in a dose dependent manner in lipid raft membrane microdomains, without significant changes in their counterparts from the cellular membranes and cytosol fractions (Fig. 2a).

To further investigate the role of G_αi_ proteins in this activation, C4-2B cells were transfected with wild type G_αi2_ (WT-G_αi2_), constitutively active G_αi_^Q205L^, or plasmid control (pcDNA3.1) (Fig. 2b). Overexpression of G_αi2_ increased basal levels of pHER2 and pSrc relative to pcDNA3.1 transfected control; these levels were further increased by CXCL12 treatment. Mutation of the activating residue of G_α_ subunit (Q205L) results in the inability of the G_α_ subunit to dissociate from GTP and is thus constitutively active. G_αi_^Q205L^ transfection resulted in high levels of pHER2 and pSrc, which were further increased by CXCL12 treatment (Fig. 2b). Overexpression of G_αi2_ also activated HER2 and Src phosphorylation in lipid raft membrane microdomains (Fig. 2c). These results show that activation of G_αi_ is sufficient for HER2 and Src phosphorylation, and that, in cancer cells, activation of small G proteins can activate downstream pathways including HER2 and Src. To determine the impact of G_αi_ protein activation of HER2 and Src on cellular invasion, C4-2B cells were treated with PTX and matrigel invasion assays were performed. CXCL12 induced cellular invasion of C4-2B cells due to the activation of Src and HER2 signaling. Further, PTX pretreatment inhibited both basal and CXCL12 invasion due to the inhibition of trimeric G-protien activation and subsequent suppression of Src and HER2 activation (Fig. 2d). Expression of G_αi_^Q205L^ enhanced basal invasion and CXCL12 treatment further enhanced cellular invasion (Fig. 2e). Together, these data demonstrate that G protein signaling mediates HER2 and Src kinase activation, and this activation may contribute to cellular invasion downstream of the CXCL12/CXCR4 axis.

### Src inhibition decreases CXCL12-mediated invasion

**Fig. 3:**
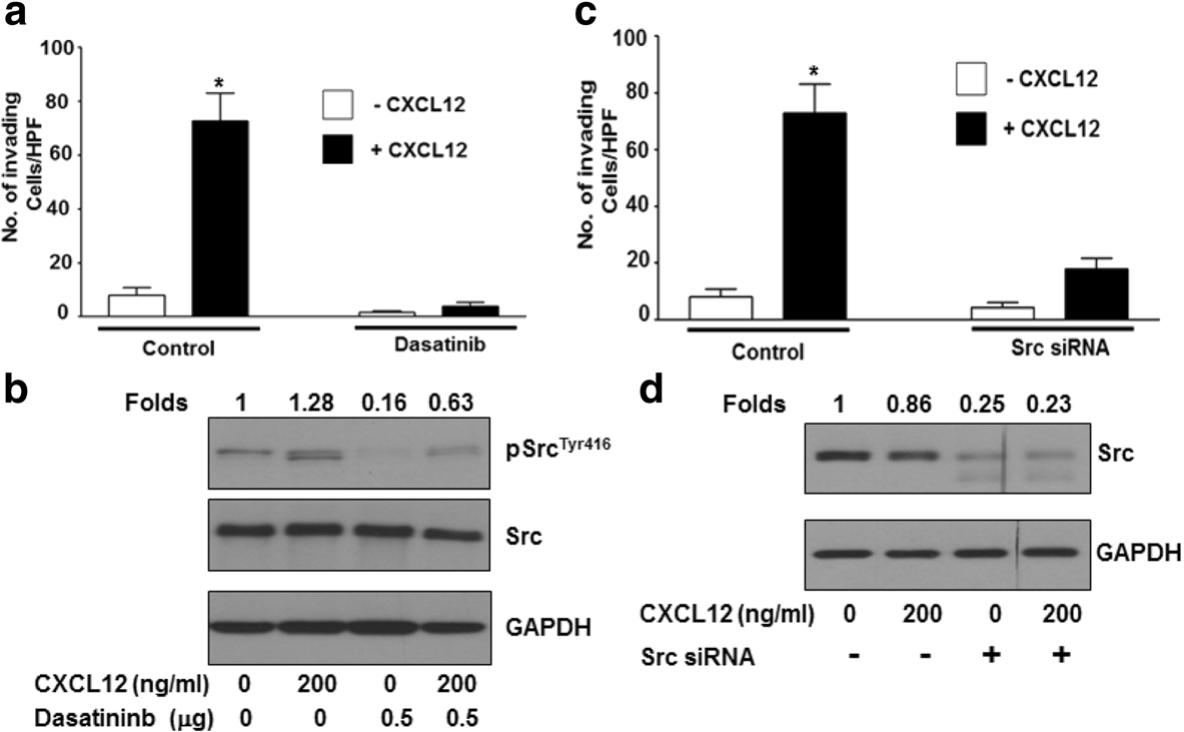
Src inhibition abrogates CXCL12 induced C4-2B cell invasion. **a** C4-2B cells were plated in serum free medium in the presence or absence of 0.5 μM Dasatinib on Matrigel coated inserts with or without 200 ng/mL CXCL12 in the bottom well and allowed to invade for 24 h. **b** C4-2B cells were plated in serum free medium in the presence or absence of 0.5 μM Dasatinib for 24 h. Prior to cell lysate preparation cells were challenged with 200 ng/mL CXCL12 for 15 min. Protein expression of phosphorylated Src, total Src and GAPDH was determined by Western blot analysis, and fold changes were calculated based on densitometric scanning of bands in X-ray film. **c** C4-2B cells were transfected with control or Src siRNA; after 24 h, cells plated in serum free medium on Matrigel coated inserts with or without 200 ng/mL CXCL12 in the bottom well were allowed to invade for 24 h. In both invasion experiments (**a** and **c**), the number of invading cells was quantitated in three independent experiments, statistical analyses were performed using ANOVA, and significance was calculated using Tukey posttest analysis using GraphPad Prism software. **d** C4-2B cells were transfected with control or src siRNA; after 24 h cells were cultured in the presence or absence of 200 ng/mL CXCL12 for 15 min in serum free medium. Cell lysate was collected after 24 h. Protein expression of total Src and GAPDH was determined by Western blot analysis, and densitometry was performed (numbers indicate fold change, normalized to untreated controls)

The previous results implicate G proteins in the activation of Src. To determine the downstream effects of this activation, the role of Src in CXCL12-mediated invasion was next investigated. To this end, dasatanib was used to inhibit Src as a pharmacological approach and Src siRNA was used as a genetic approach. Dasatanib treatment of C4-2B cells leads to inhibition of basal and CXCL12 induced matrigel invasion (Fig. 3a). Western blot analysis confirmed the inhibition of Src phosphorylation by dasatinib (Fig. 3a). To confirm the role of Src in CXCL12-mediatied invasion, Src expression in C4-2B cells was downregulated using siRNA 24 h prior to plating on Matrigel-coated inserts. As shown in Fig. 3c, Src siRNA resulted in a decrease in the CXCL12-mediated invasion. Western blot analysis confirmed the downregulation of Src by siRNA (Fig. 3d). These results confirm that, upon transactivation of Src through CXCL12/CXCR4, Src is capable of mediating downstream effects such as invasion.

### Inhibition of CXCR4 decreases initial establishment of bone tumors without affecting the expansion of existing bone tumors

**Fig. 4:**
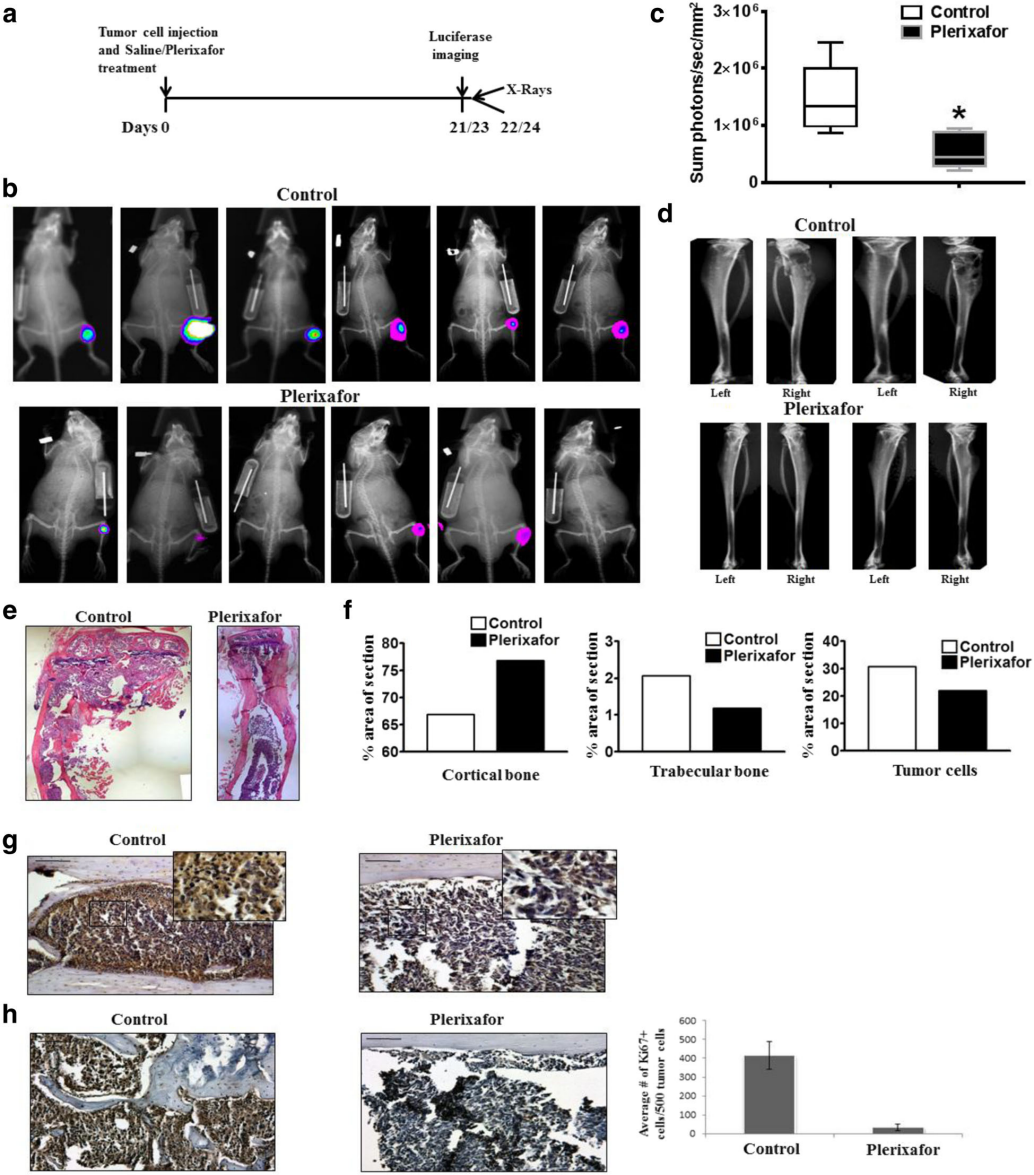
Treatment with plerixafor results in decreased intratibial tumor growth when administered concurrently with tumor cell injection. **a** Diagram of experiment timeline. On Day 0, PC-3 M-luc2 cells were injected intratibially and saline control or plerixafor treatment was started via an osmotic pump. **b** After 21 or 23 days, in vivo luciferase imaging was performed. Images shown are taken at either 21 or 23 days. **c** Tumor growth at 21 or 23 days post injection of cells was determined by in vivo luciferase imaging (*n* = 6). * Represents *p* < 0.05. **d** Ex vivo x-ray imaging of media injected (left) and tumor bearing (right) tibiae was performed at 22 and 24 days post-injection. **e** H&E was performed on tissue sections from PC-3 M-luc2 intratibial tumors treated with saline control or plerixafor. Representative images were taken at 5X and digitally merged. **f** H&E staining was used for bone histomorphometric analysis to determine the ratio of cortical bone (*left*), trabecular bone (*middle*), and tumor cells (*right*) in each tissue section. **g** Tissue sections from PC-3 M-luc2 intratibial tumors were immunostained with anti-CXCR4 antibody. Images were taken at 20X and 40X (insert). **h** Proliferation of PC-3 M-luc2 tumors was analyzed by Ki67 staining; an average number of Ki67+ cells from five 20X fields was determined

To determine the biological significance of the CXCL12/CXCR4 axis in bone tumor growth, an intratibial model was employed. In this model system, CXCR4 inhibition was obtained by use of plerixafor. Plerixafor is a hematopoetic stem cell mobilizer that has been shown to block the binding of CXCL12 to CXCR4. To this end, PC-3 M-luc2 cells were used to test the inhibition of CXCR4 signaling with plerixafor. Western blot analysis showed that PC-3 M-luc2 cells express higher CXCR4, but similar levels of EGFR, compared to PC-3 cells (Additional file 1: Figure S1). PC-3 M-luc2 cells were injected intratibially and saline control or plerixafor treatment was started via an osmotic pump on day 0 (Fig. 4a). After 21 or 23 days, in vivo luciferase imaging was performed. Twenty-two or twenty-four days post-injection of tumor cells and initiation of treatment, mice were sacrificed and ex vivo x-ray imaging was performed on the harvested tibiae. Treatment of the mice with plerixafor resulted in decreased tumor growth and decreased bone destruction relative to the saline control, as shown by luciferase and x-ray imaging (Fig. 4b-d). Bone histomorphometry performed on bone tissue sections revealed that plerixafor treatment resulted in decreased tumor volume and a decrease in bone destruction (Fig. 4e, f). Immunohistochemical analysis demonstrated reduced expression of CXCR4 in the plerixafor treated mice (Fig. 4g). Additionally, treatment with plerixafor resulted in decreased proliferation, as determined by Ki67 staining (Fig. 4h). Plerixafor also inhibited tumor growth and tumor induced bone destruction in a C4-2B-luc2 model (Additional file 1: Figure S2). These results demonstrate that inhibition of CXCR4 at the time of prostate tumor implantation in the bone results in decreased tumor growth, which is associated with decreased CXCR4 expression, decreased tumor induced bone osteolysis, and decreased proliferation.

**Fig. 5:**
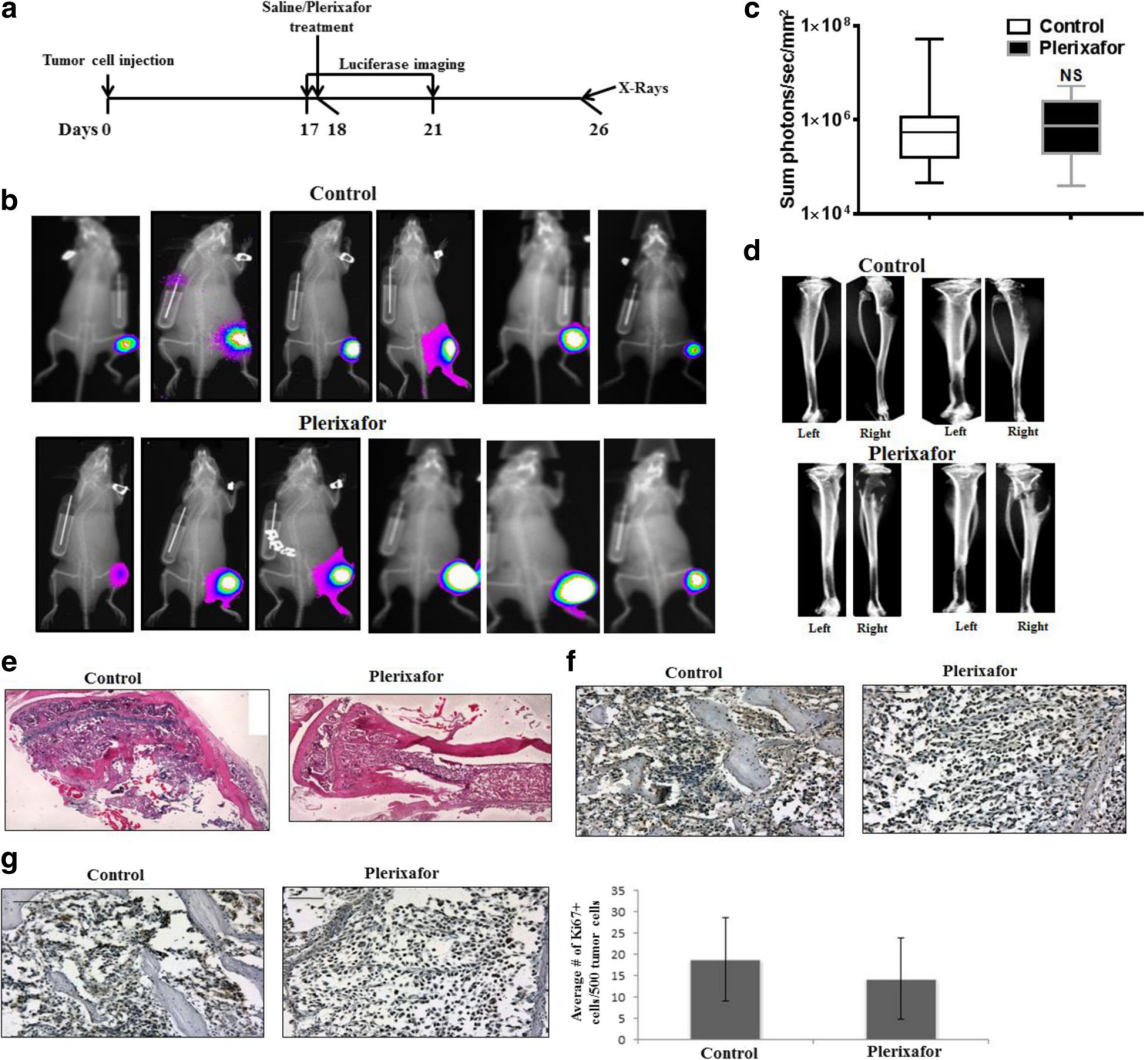
Treatment with plerixafor does not alter intratibial tumor growth when administered subsequent to tumor formation. **a** Diagram of experiment timeline. On Day 0, PC-3 M-luc2 cells were injected intratibially. After allowing tumors to grow for 18 days, saline control or plerixafor treatment was started via an osmotic pump. **b** After 21, days post-injection of tumor cells, in vivo luciferase imaging was performed. **c** Tumor growth at 21 days post injection of cells was determined by in vivo luciferase imaging (*n* = 10). NS represents no significant difference between groups. **d** Ex vivo x-ray imaging of media injected (left) and tumor bearing (right) tibiae was performed at 26 days post-injection. **e** H&E was performed on tissue sections from PC-3 M-luc2 intratibial tumors treated with saline control or plerixafor. Representative images were taken at 5X and digitally merged. **f** Tissue sections from PC-3 M-luc2 intratibial tumors were immunostained with anti-CXCR4 antibody. **g** Proliferation of PC-3 M-luc2 tumors was analyzed by Ki67 staining; the average number of Ki67+ cells from five 20X fields was calculated

To determine if inhibition of CXCR4 would have similar effects on tumor growth with established tumors, mice were again injected intratibially with PC-3 M-luc2 cells. After allowing tumor growth for 18 days, mice were divided into two groups with relatively equal luciferase signal. Osmotic pumps were implanted the next day with either plerixafor or saline. Tumor growth was monitored by in vivo imaging at 17 and 21 days post-injection of the cells. Mice were sacrificed 26 days after tumor cell injection, and ex vivo imaging of the tibiae was performed (Fig. 5a). As shown by luciferase imaging as well as by x-ray scans and histology, treatment with plerixafor at this stage did not affect tumor growth relative to the saline control (Fig. 5b-e). Immunohistochemical analysis of bone sections did not reveal a change in CXCR4 expression upon plerixafor treatment (Fig. 5f). Additionally, plerixafor treatment did not affect proliferation, as determined by Ki67 (Fig. 5g). These results demonstrate that inhibition of CXCR4 subsequent to prostate tumor development in the bone does not affect tumor growth. Taken together with the results of Fig. 4, it is likely that CXCL12/CXCR4 signaling plays a key role in the initial establishment of prostate tumors in the bone; however, the importance of CXCL12/CXCR4 appears to be taken over by other signaling mechanisms during the enlargement phase of bone tumors.

### EGFR and HER2 inhibition blocked the expansion of bone tumor

**Fig. 6:**
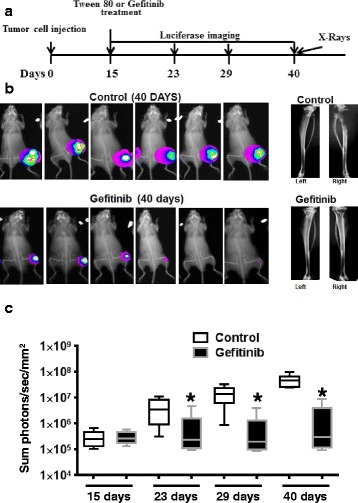
Gefitinib treatment inhibits intra-tibial tumor growth when administered subsequent to tumor formation. **a** Diagram showing the experimental scheme, where PC-3 M-luc2 cells were implanted at day 0. Luciferase imaging was performed at days 15, 23, 29 and 40. Gefetinib (200 mg/kg body weight) or placebo containing 0.5 % Tween 20 administration through gavage started at day 15. X-rays were taken at day 40. **b** In vivo luciferase images were taken at 40 days for both groups, followed by a representative x-ray picture of a set of animals in both groups. **c** Quantitation of in vivo luciferase data from *N* = 6 animals in both control and Gefinitib treated animals, starting from 15 days till the end of the experiment on the 40th day. Wilcoxon rank sum test were performed between control and experimental groups, where * represents *p* < 0.05

Our data suggest that CXCR4 induces growth factor receptor activation through EGFR and HER2 phosphorylation (Fig. 1) and subsequent expression of proteases and cellular invasion [3]. Growth factor ligands activate EGFR by direct binding, whereas HER2 is inactive in direct ligand binding but heterodimerization with ligand bound EGFR potently amplify its signaling [37]. Gefitinib is a broad EGFR family member inhibitor which has been shown to inhibit EGFR signaling and HER2 signaling through heterodimerization with EGFR [38]. Since CXCR4 transactivates EGFR and HER2, we tested whether growth factor receptor signaling contributes to bone tumor growth. Mice having established bone tumors were randomized and treated with either gefitinib or Tween 20 (formulation used for dissolving gefitinib). Tumor growth was monitored at 23, 29 and 40 days by in vivo imaging and bioptic x-rays were performed at the 40th day of post tumor cell implantation (Fig. 6a). Gefitinib treatment inhibited bone tumor growth starting from as early as 8 days after treatment and persisted till the 25th day. X-rays showed less osteolysis in gefinitib treated animals (Fig. 6b). This data suggest that EGFR and HER2 signaling is actively involved in bone tumor growth in the PC-3 M-luc2 bone tumor model.

## Discussion

The CXCL12/CXCR4 axis has been shown to be involved in metastasis of several types of cancers, including prostate cancers. In support of CXCL12/CXCR4 function in tumor metastasis, herein, we show that: i) CXCL12/CXCR4 transactivates members of growth factor receptors, EGFR and HER2 and that this transactivation is largely confined to lipid raft membrane microdomains in PC cells; ii) G_αi_ protein activation is required for downstream signaling involving HER2 and Src, and constitutively active G_αi_ proteins can activate HER2 and Src signaling, independent of CXCR4 activation; iii) Src activation mediates CXCL12/CXCR4 induced cell invasion; iv) plerixafor is effective in inhibition of tumor growth, when given at the time of tumor implantation, but not effective against established tumors, suggesting the CXCL12/CXCR4 axis is crucial for initial interaction with bone microenvironment and that this interaction dictates subsequent growth of bone tumors; and v) the pan EGFR family member inhibitor gefitinib inhibited growth of established bone tumors, suggesting that growth factor signaling is critical for the expansion/enlargement of bone metastases.

Lipid rafts are specialized entities in plasma membrane, enriched with tightly packed saturated lipids and cholesterol which gives liquid ordered states. These raft microdomains are known to segregate different constituents on the membrane thereby facilitating signal transduction. Previous studies show that CXCR4 signaling localizes to lipid raft membrane microdomains and that disruption of lipid rafts leads to inhibition of basal and CXCL12 signaling [3, 6]. The association of CXCR4 with lipid rafts in hematopoietic stem cells was shown to promote bone marrow retention and regulate homing as well as mobilization of hematopoietic stem cells [39]. Previous studies used detergents to isolate lipid rafts based on the fact that these rafts were insoluble in non-ionic detergents, but the use of detergents was implicated in artificial coalescence of signaling proteins. To address this issue we used detergent-free cell lysate preparations for lipid raft isolation using the sucrose density buoyant centrifugation method and show that the lipid raft marker flotillin co-sediments with CXCR4 and phosphorylated HER2 and Src kinases (Fig. 1d). Previous studies also show that EGFR family members localize to lipid raft preparations in detergent-free conditions [33, 35]. These observations suggest that CXCR4 and its signaling partners localize to lipid rafts and initiate signaling events leading to cell invasion.

G-proteins are key regulators of G-protein coupled receptor (GPCR) signaling and were shown to promote oncogenic signaling. Previous studies show that activated forms of G12 and G13 promote PC cell invasion, and G_αi_ proteins were also shown to promote cell migration. Our data show that PTX abrogated both HER2 and Src phosphorylation and cellular invasion, suggesting that G_αi_ proteins are indispensable for CXCR4 induced cellular invasion. Moreover, we show that constitutively activated G_αi_ protiens are sufficient to mediate HER2 and Src phosphorylation and this phosphorylation may contribute to cellular invasion of PC cells, suggesting that CXCR4 activated G_αi_ proteins are sufficient for PC cell invasion. Recent studies support the role of G_αi_ proteins in Src induced formation of invadosomes, which are cellular protrusions exhibited in migrating/invading cells, where a transient activation of Cdc42 is implicated downstream of GPCR activation [40]; thus, we cannot rule out the role of Cdc42 downstream of CXCR4 activated Src activation in PC cells.

CXCL12/CXCR4 signaling regulates hematopoietic stem cell (HSC) movement in and out of bone marrow. HSCs home to the CXCL12 rich endosteal niche in bone marrow, where upon entering the bone, they anchor to the niche through CXCR4 activation of α4β1 integrins with vascular cell adhesion molecule 1 (VCAM1) expressed in the niche. These chemokine (CXCL12/CXCR4) and cell adhesion molecule (VCAM1/α4β1) interactions restrain the HSCs in the bone marrow niche. Plerixafor is a stem cell mobilizer, where it competitively inhibits CXCL12 binding to CXCR4, thereby disrupting the HSC interaction with bone marrow niches, leading to exit of cells from bone marrow. Here we show that inhibition of CXCR4 with plerixafor is effective against bone tumor growth when given initially during tumor implantation. Similar results were previously documented for neutralization of CXCR4 with anti-CXCR4 antibodies in an intracardiac model of metastases where anti-CXCR4 antibodies reduced total metastatic burden, including that of long bone [29]. Recent studies suggest that initial arrival of tumor cells in the bone microenvironment leads to competition of tumor cells with HSCs for occupation at osteoblastic niches [2]. Since both HSCs and PC cells use the CXCL12/CXCR4 axis for occupying the osteoblastic niche, plerixafor inhibited CXCL12/CXCR4 interactions, leading to reduced tumor growth when given at the time of tumor implantation. Reduction in tumor growth was also accompanied by reduced tumor induced bone destruction. This suggests that plerixafor not only mediated egress of HSCs from bone but also inhibited initial interaction of tumor cells with the osteoblastic niche, leading to inhibition of tumor growth. In support of our observation, while this manuscript was in preparation, Gravina et al. showed that plerixafor inhibited intratibial tumor growth when administered right after tumor cell implantation [41]. As the putative PC stem cells use the CXCR4 pathway for metastasis and chemotherapies often are effective in eliminating these cell populations [42], plerixafor mediated inhibition of CXCR4 may inhibit bone metastasis by these cell populations.

Plerixafor administration to established tumors did not significantly impact tumor growth, suggesting that, at high tumor burden, plerixafor mediated egress of tumor cells from bone, as suggested by previous studies [2], is overcome by the growth signals in tumors. CXCL12/CXCR4 transactivates members of growth factor receptor in PC cells [3, 6], and expression of HER2 in PC patients correlates with tumor cell proliferation [43] and activates androgen receptor signaling in advanced disease [44]. To address the potential role of growth factor receptor activation contributing to the bone tumor growth, we treated established bone tumors with gefitinib and found our data (Fig. 6) support the notion that inhibition of growth factor receptor mediated signals with gefitinib led to reduction in tumor burden and tumor induced osteolysis in bone tumors. It remains to be determined that combination of the two therapies have an added effect over individual therapies, but based on the mechanism of target inhibition combination therapy may provide a more efficacious inhibition of bone tumor growth.

Taken together, CXCL12/CXCR4 signaling has a dual impact in bone metastasis: newly arrived cancer cells in bone use CXCL12/CXCR4 and integrin interactions to localize to the endosteal niche, and established bone tumors use CXCL12/CXCR4 transactivated growth factor receptor signaling for expansion of bone tumors.

## Conclusions

CXCL12/CXCR4 transactivation of members of growth factor receptors exclusively occurs in lipid raft membrane microdomains. G_αi_ protein activation is required for downstream signaling involving EGFR, HER2 and Src in lipid raft membrane microdomains. Plerixafor, a competitive CXCR4 inhibitor and a stem cell mobilizer, is effective in inhibiting initial establishment of tumor cells into the bone microenvironment, whereas the same drug is ineffective in containing the expansion of pre-existing bone metastasis. Interestingly, in our model system, the growth factor receptor inhibitor gefitinib is highly effective against expanding bone tumors. Based on our preclinical observations, plerixafor may be a candidate drug for the patient populations which are high risk for developing metastasis, with low metastasis burden, and who are prone to relapse, whereas gefitinib may be a candidate for patients with metastatic disease with rising PSA. Further experiments are in progress to determine the efficacy of different drug regimens (sequence of gefitinib and plerixafor) with or without chemotherapy combination for treating established bone tumors.

## Additional file

Additional file 1: Figure S1. CXCR4 and EGFR expressed in PC-3 M-luc2 cells. Western blot analysis of EGFR and CXCR4 shows that PC-3 M-luc2 cells have higher CXCR4 expression compared to PC3 and C4-2B. No significant change in EGFR expression was observed in these cells. **Figure S2.** Plerixafor treatment inhibited intratibial tumor growth in C4-2B-luc cells. A) Diagram of experimental timeline. On Day 0, C4-2B-luc cells were injected intratibially and saline control or Plerixafor treatment was started via an osmotic pump. B) After 12, 19, 26 and 33 dyas, in vivo luciferase imaging was performed. Images shown are taken at 33 days. Ex vivo x-ray imaging of media injected (left) and tumor bearing (right) tibiae was performed at 23 days post-injection C) Tumor growth at 12, 19, 26 and 33 days post injection of cells was determined by in vivo luciferase imaging. (PPTX 659 kb)

## Abbreviations

Akt kinase: Autologous killer t-cell kinase
ATCC: American type culture collection
BCA: Bicinchoninic acid
C4-2B: A prostate cancer cell line derived from LNCaP cells
Cdc42: Cell division control protein 42 homoolog
CXCL12: Chemokine ligand 12 containing CXC motif
CXCR4: Chemokine receptor 4, whose ligand is CXC motifcontaining chemokine
EGFR: Epidermal Growth Factor Receptor
ERG: A member of Ets transcription family
EtOH: Ethanol
FBS: Fetal bovine serum
GAPDH: Glyceraldehyde phosphate dehydrogenase
GTP: Guanine nucleotide triphosphate
G_αi2_: Inhibitory trimeric G-protein alpha 2 isoform
HER2: Heregulin Epidermal growth factor Receptor 2
HSC: Hematopoietic stem cell
Ki67: A cellular marker for proliferation
Luc: Luciferase
NF-κB: Nuclear factor kappa B
PC: Prostate cancer
PC3: A prostate cancer cell line
PC-3 M-luc2: A metastasis PC3 cell line expressing luciferase gene
PMSF: Phenylmethysulfonyl fluoride
PTX: Pertussis toxin
RPMI: A cell culture media developed at Roswell Park Memorial Institute
Scr: Scrambled
SE: Standard error
siRNA: Small inhibitory RNA
Src: Non-receptor tyrosine kinase (Src is an abbreviation for sarcoma)
STR: Short tandem repeat
TMPRSS2: Transmembrane serine proteinase 2
VCAM: Vascular cell adhesion molecule
VCaP: A prostate cancer cell line
α4β1: An integrin protein consisting of alpha 4 and beta 1 polypeptide chains
μg: Microgram
