# Supplementary material for: Pharmacological targeting of CXCL12/CXCR4 signaling in prostate cancer bone metastasis
Source: Mol Cancer. 2016 Nov 3;15:68. doi: 10.1186/s12943-016-0552-0 (PMC5093938; doi:10.1186/s12943-016-0552-0)
Supplement: Additional file 1: Figure S1. — CXCR4 and EGFR expressed in PC-3 M-luc2 cells. Western blot analysis of EGFR and CXCR4 shows that PC-3 M-luc2 cells have higher CXCR4 expression compared to PC3 and C4-2B. No significant change in EGFR expression was observed in these cells. Figure S2. Plerixafor treatment inhibited intratibial tumor growth in C4-2B-luc cells. A) Diagram of experimental timeline. On Day 0, C4-2B-luc cells were injected intratibially and saline control or Plerixafor treatment was started via an osmotic pump. B) After 12, 19, 26 and 33 dyas, in vivo luciferase imaging was performed. Images shown are taken at 33 days. Ex vivo x-ray imaging of media injected (left) and tumor bearing (right) tibiae was performed at 23 days post-injection C) Tumor growth at 12, 19, 26 and 33 days post injection of cells was determined by in vivo luciferase imaging. (PPTX 659 kb) [file 12943_2016_552_MOESM1_ESM.pptx]

## Slide 1
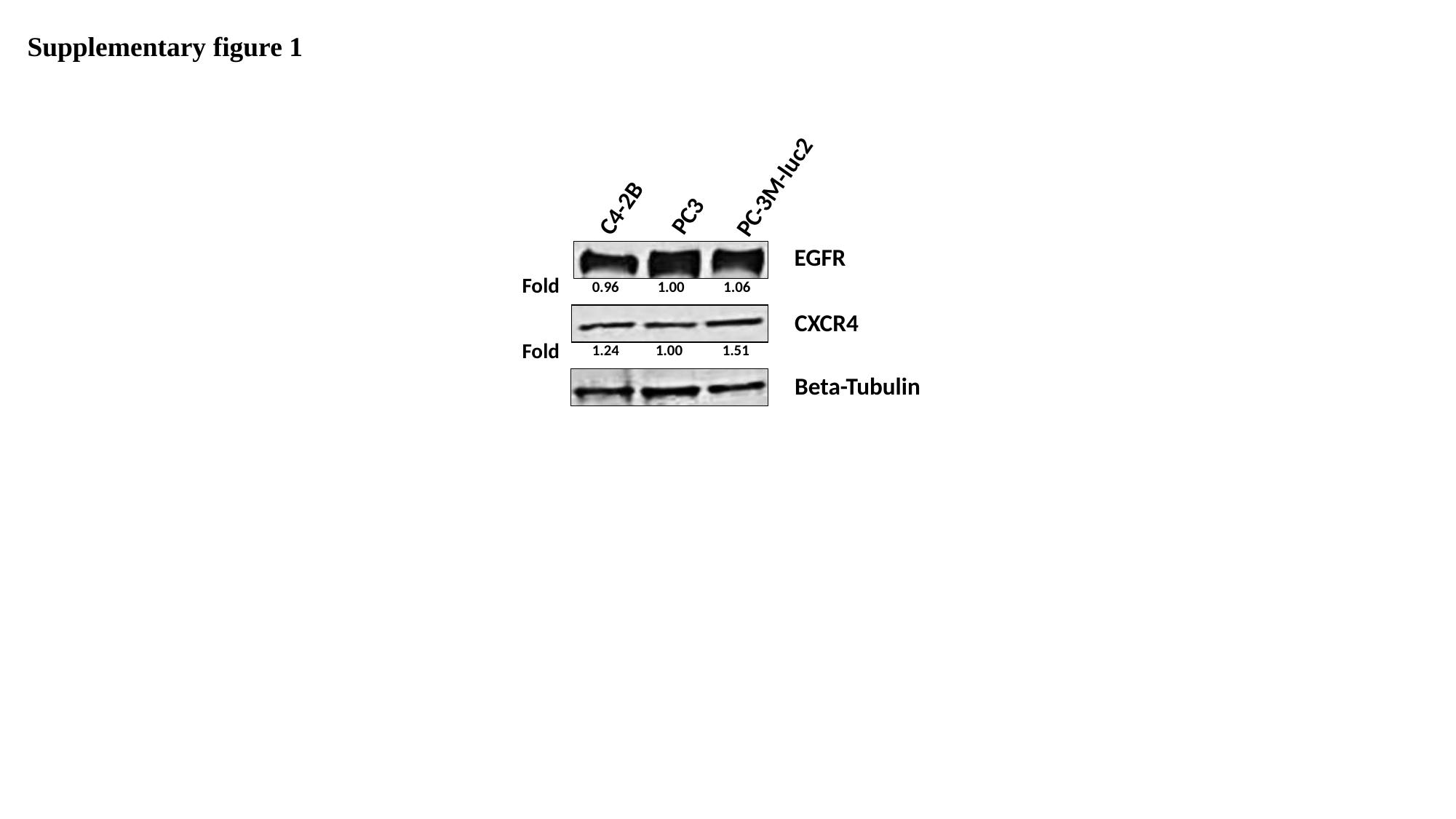

Supplementary figure 1
PC-3M-luc2
C4-2B
PC3
EGFR
Fold
0.96
1.00
1.06
CXCR4
Fold
1.24
1.00
1.51
Beta-Tubulin

## Slide 2
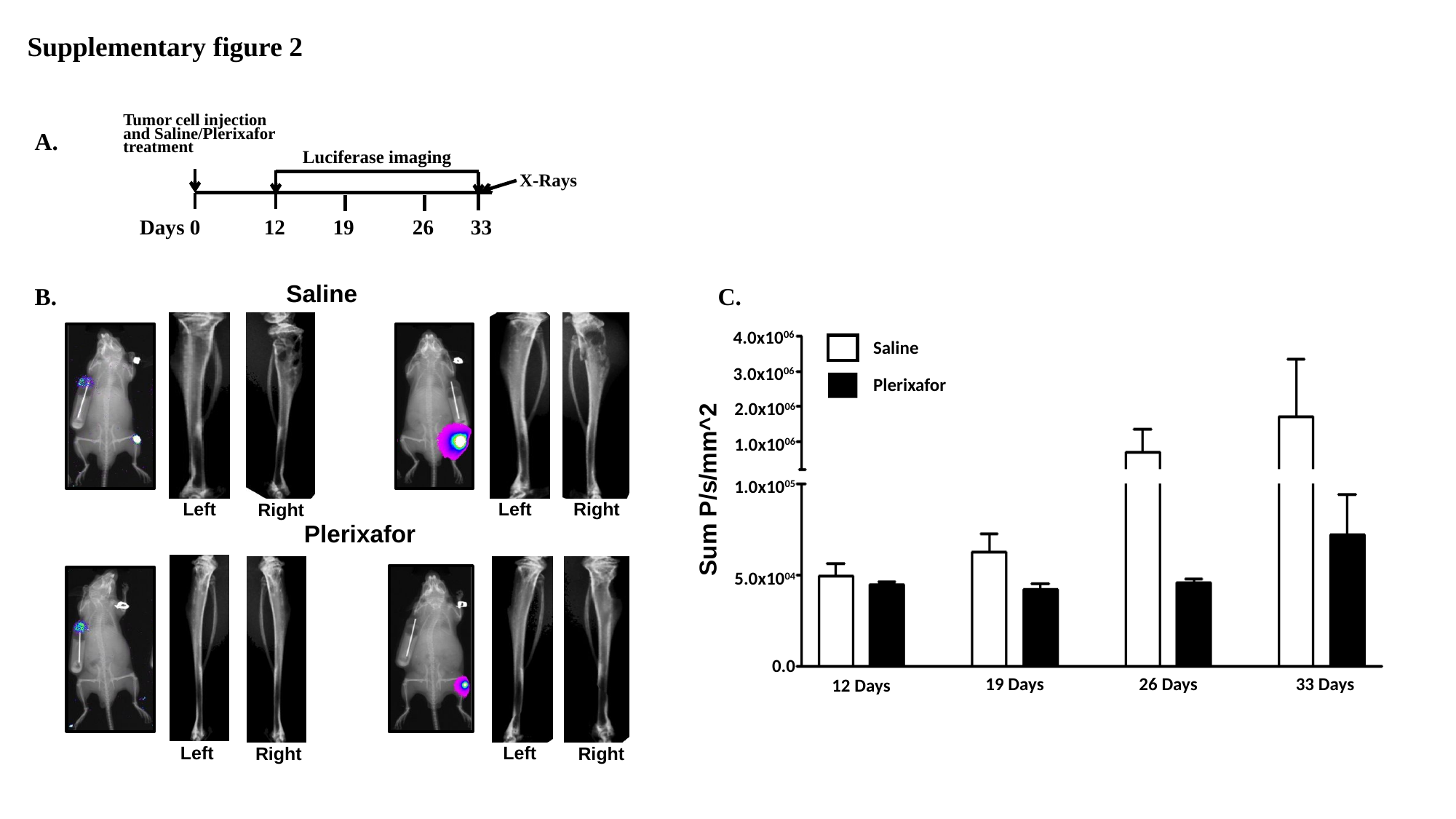

Supplementary figure 2
Tumor cell injection
and Saline/Plerixafor
treatment
A.
Luciferase imaging
X-Rays
 Days 0 12 19 26 33
Saline
Left
Left
Right
Right
Plerixafor
Left
Left
Right
Right
B.
C.
4.0x1006
Saline
3.0x1006
Plerixafor
2.0x1006
1.0x1006
1.0x1005
Sum P/s/mm^2
5.0x1004
0.0
19 Days
26 Days
33 Days
12 Days
